# Application of a novel rectal cooling device in hypothermia therapy after cerebral hypoxia-ischemia in rats

**DOI:** 10.1186/s12871-016-0239-5

**Published:** 2016-09-09

**Authors:** Peng Liu, Rui Yang, Zelan Zuo

**Affiliations:** 1 Department of PICU, Children’s Hospital of Chongqing Medical University, Chongqing, 400014 China; 2 Ministry of Education Key Laboratory of Child Development and Disorders, China International Science and Technology Cooperation Base of Child Development and Critical Disorders, Chongqing Key Laboratory of Pediatrics, Chongqing, 400014 China

**Keywords:** Therapeutic hypothermia, Rectal cooling device, Cell apoptosis

## Abstract

**Background:**

A new rectal cooling device for therapeutic hypothermia (TH) therapy is designed and is applied in TH treatment of SD rats with ischemic-hypoxic brain damage.

**Methods:**

Healthy adult SD rats (*n* = 45) were randomly assigned into four groups: the healthy control group (*n* = 5), the ischemia and hypoxia group (*n* = 10), the rectal TH cooling group (*n* = 18), and the ice blanket TH cooling group (*n* = 11). The rats in the rectal cooling and ice blanket TH groups received 12 h treatment after hypoxic-ischemic brain damage had been established, while those in the ischemia and hypoxia group did not. Taking the start of TH as the zero point, rats were sacrificed after 24 h and the brain and rectum tissues were sampled for histological analysis.

**Results:**

The TH induction time (37.3 ± 14.7 min) in the rectal cooling group was significantly shorter (F = 4.937, *P* < 0.05) than that in the ice blanket cooling group (75.6 ± 27.2 min). The HE and NISSL staining results showed that rats in the rectal TH cooling group had significantly decreased (*P* < 0.01) positive neurons cell count compared to those in ischemia and hypoxia group. In addition, TUNEL staining indicated that the number of apoptotic cells (3.9 ± 1.8 cells / × 400 field) and the apoptosis index (4.4 % ± 1.5) were significantly lower in rectal TH cooling group (*P* < 0.05) than in ischemia and hypoxia group (23.2 ± 12.1 cells / × 400 field, 26.6 % ± 12.1). Also, no rectal frostbite or inflammatory infiltration was observed in rats in the rectal TH treatment groups.

**Conclusion:**

Our new cooling device realized rapid TH induction in SD rats with ischemic-hypoxic brain damage, inhibited the apoptosis of cells in the hippocampal CAl region, and did not cause histological damage to the rectal tissues.

**Electronic supplementary material:**

The online version of this article (doi:10.1186/s12871-016-0239-5) contains supplementary material, which is available to authorized users.

## Background

Therapeutic hypothermia (TH) therapy plays a significant role in the functional protection of brain, heart, liver, kidney, and other vital organs in patients undergoing cardiopulmonary resuscitation or suffering from hypoxic-ischemic brain damage and multiple organ failures [1–5]. When neonatal suffering from hypoxic-ischemic brain damage,which also called neonatal hypoxic-ischemic encephalopathy, many neonatal center have established hypothermia as the only effective treatment available [6–8]. In those patients TH therapy could improve cellular energy metabolism; decrease basal metabolic rate of tissues and organs, consumption of oxygen and energy, generation of oxygen radicals and intracellular calcium overload; improve cell necrosis and apoptosis; promote the recovery of intercellular signaling; alleviate cerebral edema and reduce intracranial pressure. TH therapy could significantly improve patient’s quality of life and lessen the rate of disability or mortality. The earlier the therapy is given, the more significant the protective effect will be.

Therapeutic hypothermia (TH) is defined as a core body temperature of 28–35 °C. The temperature controlled within 32–34 °C is often considered safe and, therefore, is most commonly used for the TH therapy on experiment animals. This is because, within this range, the blood pressure, blood oxygen, carbon dioxide partial pressure, blood pH and blood glucose would not be influenced, and no pathological damages would occur in animals’ heart, lung, kidney, small intestine and other organs.

Nowadays, a variety of methods could be used for TH cooling, and the cooling devices have also undergone continuous improvement and development. Reported methods for TH cooling include ice bag cooling, ice blanket cooling, intravenous infusion of a low-temperature liquid, extracorporeal blood cooling, intravascular catheter cooling, blood filtration, and selective head cooling [9–15]. Some TH therapy devices introduced by top hospitals are too expensive for hospitals in small counties or villages to afford, and too large to move, thus limiting their application in prehospital TH treatment. These shortcomings significantly inhibit the promotion of TH therapy, and may even delay the treatment opportunity for critically sick patients.

With the increasingly wide applications of TH therapy, how to develop a fast and economical cooling device has become one research hot spot. In this study, a new rectal cooling device for therapeutic hypothermia (TH) therapy was designed and used to treat SD rats with ischemic-hypoxic brain damage, which serves as an experimental basis for future clinical trials.

## Methods

### Experimental animals

Healthy SD rats (male and female), weighing 250–300 g were provided by the Laboratory Animal Center of Chongqing Medical University.

### The design of a new rectal TH cooling device (Fig. 1)

**Fig. 1:**
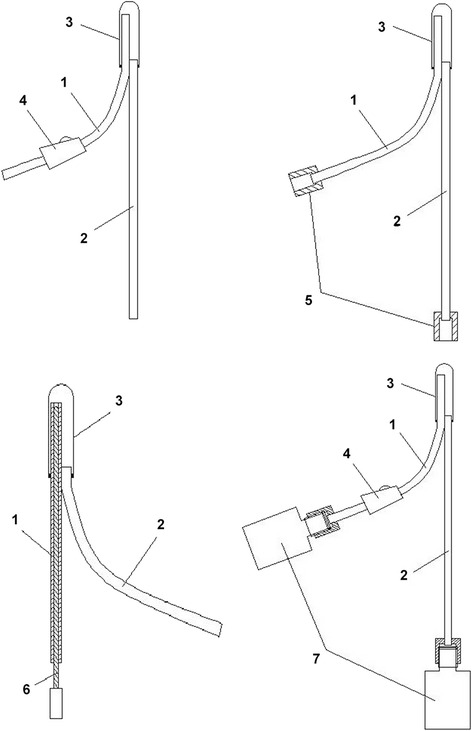
Schematic diagram of the rectal TH cooling device 1: The inflow tube; 2: the outflow tube; 3: The hydration bladder; 4: The speed controller; 5: The connector: Connectors could be linked to common infusion sets, and different connectors could be designed according to study needs; 6: The guiding wire: The guiding wire is used to insert the hydration bladder into the rectum of rats; 7: The removable liquid bag: The bag functions for liquid storage and is connected to both the inflow and outflow tubes. An insulation bag was designed for the bag connected to the inflow tube. Different insulation bags could be designed according to study needs

The inserted inflow and outflow tubes have different lengths. The end of the inflow tube is inserted into the bottom of the hydration bladder, and the end of the outflow tube is inserted to a depth approximately at the anal entrance. Low-temperature liquid flows into the hydration bladder via the inflow tube and flows out via the outflow tube. The hydration bladder is closed at the bottom and functions only for liquid storage. The length of the bladder is equivalent to that of the rectum (5 cm).

In this study, 0 °C ice-water mixture was used as a low-temperature infusion circulation liquid. A infant transfusion needle without the needle handle was used as the inflow and outflow tubes. A balloon was used as the hydration bladder. The speed controller and connector used for circulation were also used in this device. The guiding wire provided with the urethral catheterization pack was used for inserting the hydration bladder. 500-mL or 1000-mL infusion bag was used as the liquid storage bag. Two temperature probes were respectively connected to the inflow and outflow tubes for detecting the temperature of the water at two ends, thereby evaluating the heat exchange effect and preventing excessively low temperature.

### Animal grouping

Healthy adult SD rats (*n* = 51) were randomly assigned into four groups: the healthy control group (*n* = 5), the ischemia and hypoxia group (*n* = 10), the rectal TH cooling group (*n* = 18), and the ice blanket (HICO-VARIOTHERM550,German) TH cooling group (*n* = 11). The rats in the ischemia and hypoxia group, rectal TH cooling group and ice blanket TH cooling group had induced hypoxic-ischemic brain damage. The rats in the rectal cooling and ice blanket TH groups were treated with therapeutic hypothermia later, but those in the ischemia and hypoxia group received no treatment. The rats in the healthy control group were normal healthy rats without any form of treatment.

### Anesthesia

Wang et al. [16] suggested that studies concerning cerebral temperature variation or therapeutic hypothermia should choose anesthetic methods and agents having less influence on brain temperature. Our pilot study showed that intraperitoneal anesthesia had a greater influence on experiment animals; thus rectal administration of chloral hydrate was used in this study. Before TH treatment, SD rats were weighed and anesthetized via rectal infusion of 10 % chloral hydrate (0.3 mL/kg). During TH treatment, animals were sedated with 10 % chloral hydrate (0.1 mL/kg) every 2 h.

### Establishment of an ischemia and hypoxia model

The hypoxic-ischemic brain damage model was constructed based on the method reported by Rice et al. [17]. After anesthetizing rats with 10 % chloral hydrate, the left common carotid artery was isolated and ligated. Then, rats were returned to recover for 2 h in their cages. Subsequently, they were placed into a hypoxia box with an air flow of 8 % O_2_ and 92 % N_2_ mixture at a rate of 2 L/min for 2 h. Rats showing left-side rotation were considered as successful hypoxic-ischemic brain damaged models.

### TH treatment

#### Induction of TH

Rats in the rectal TH cooling group were anesthetized and connected to the rectal cooling device with the infusion rate at 160 dtt/min. Rats in the ice blanket TH cooling group were anesthetized and placed on the blanket (HICO-VARIOTHERM 550,Germany). The temperature of the blanket was set at 15 °C. Rats’ tympanic temperature was measured every 15 min and the time needed to reach TH (35 °C) was also recorded.

#### Maintenance of TH

After TH induction, rats’ tympanic temperature was maintained at 33–35 °C for 12 h by adjusting the speed of water inflow or the temperature of the blanket. From the start of TH induction to the first 6 h of TH maintenance, rats’ tympanic temperature was measured every 30 min and during the next 6 h of TH maintenance, the temperature was measured every 1 h. Far Infrared treatment was given if the tympanic temperature fell to the lower limit (33 °C), but the circulation of ice-water and TH treatment were maintained.

#### Temperature recovery

After TH treatment, rats were returned to their cages for steady temperature recovery. The room temperature was maintained within 25–26 °C. During the recovery time, the tympanic temperature of rats should also be monitored to ensure the increasing rate of temperature was lower than 0.5 °C/h and the total time for recovery was ≥ 5 h. This approach could protect rats from rebound hypercalcemia and hypovolemic shock induced by rapid temperature recovery.

Taking the beginning of TH as the zero point, the rats were sacrificed and their brain sampled after 24 h. The rectal tissue was taken for HE staining to identify frostbite and inflammatory infiltration.

### Determination of cell apoptosis

Since TUNEL staining could not distinguish necrotic and apoptotic cells, this study used three different staining methods (TUNEL, HE, and NISSL staining) to determine the causes of cell death. Cells that tested positive to TUNEL staining, presented with round or oval-shaped dark purple-colored chromatin (≥2) and intact nuclear membrane after HE and NISSL staining were considered as apoptotic cells. On the other hand, cells that displayed eosinophilic cytoplasm or nuclear rupture, and dispersed chromatin were considered as necrotic cells. Normal cells should show negative TUNEL staining, uniform HE/NISSL staining, and intact nuclear membrane.

Under a high-power microscope, 10 high-power fields (×400) in the hippocampal CAl region were selected to count neuronal cells positively or negatively stained by different reagents. The apoptosis index (AI) was calculated by AI = TUNEL-positive cell count / (TUNEL-positive cell count + TUNEL-negative cell count) × 100 %.

### Statistical analysis

Data is presented as means ± SD. The software SPSS 17.0 was used for statistical analysis. Inter-group comparison was made by ANOVA. *P* < 0.05 was defined as statistical significance.

## Results

### The efficiency of TH induction

**Fig. 2:**
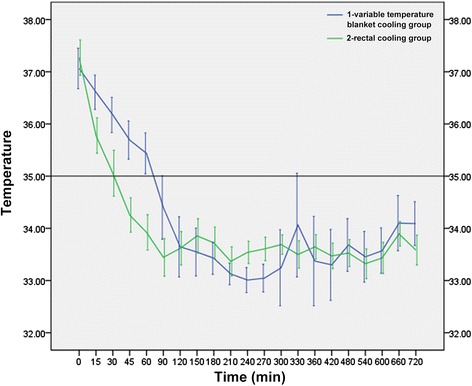
The efficiency of TH induction for the rectal cooling group and the variable temperature blanket cooling group (see more detail in Additional file 1)

During TH induction, the time needed to reach TH (35 °C) in the rectal cooling group was 37.3 ± 14.7 min, which was significantly shorter (*F* = 4.937, *P* < 0.05) than that in the variable temperature blanket cooling group (75.6 ± 27.2 min) (Fig. 2). During TH maintenance, the tympanic temperature of rats in both groups was maintained within 33–35 °C. Thanks to careful monitoring, no rats were sacrificed for temperature recovery-induced side effects.

### HE staining of rectal mucosa

**Fig. 3:**
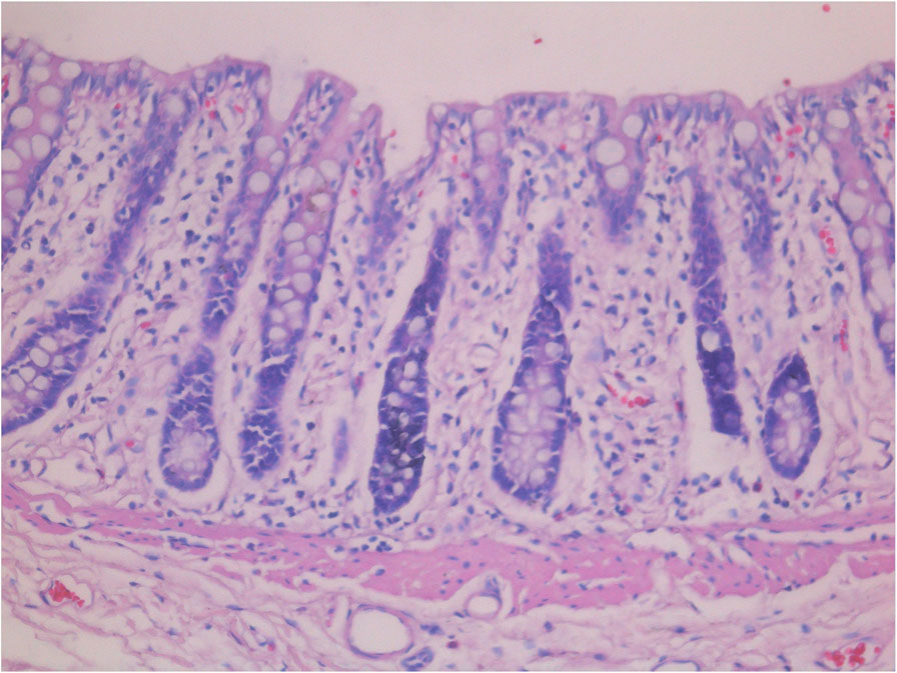
Rectal longitudinal section of healthy control HE × 400

**Fig. 4:**
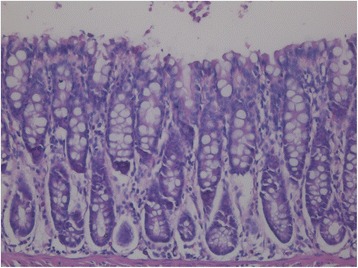
Rectal longitudinal section of rats that underwent rectal cooling HE × 400

24 h after rectal cooling, no frostbite or inflammatory infiltration was observed (Figs. 3 and 4).

### HE staining in the hippocampal CAl region

Rats in the ischemia and hypoxia group displayed significant cell damage with decreased pyramidal cell count, reduced cell size, nuclear pyknosis and cell disorder. However, rats in the rectal TH treatment group showed significantly increased number of pyramidal cells (Table 1).

**Table 1:** Positive neuron of HE staining in hippocampus CA1 (mean ± SD)

Group	Number	Positive neurons cell count in hippocampus CAl (cells/× 400 field)
Healthy control	5	0.00 ± 0.00
Ischemia and hypoxia group	10	32.48 ± 7.56
Rectal TH cooling group	18	6.30 ± 3.46

*P1* < 0.01 compared with the healthy control; *P2* < 0.01 compared with the ischemia and hypoxia group

### NISSL staining in the hippocampal CAl region

Rats in the ischemia and hypoxia group had significantly decreased number of pyramidal cells in the CAl region, while rats in the rectal TH cooling group had increased pyramidal cells, but the level was still lower than that of the healthy control group (Table 2).

**Table 2:** Positive neurons of NISSL staining in hippocampus CA1 (mean ± SD)

Group	Number	Positive neurons cell count in hippocampus CA1 (cells/× 400 field)
Healthy control	5	0.00 ± 0.00
Ischemia and hypoxia group	10	20.08 ± 5.40
Rectal TH cooling group	18	4.90 ± 2.20

*P1* < 0.01 compared with the healthy control; *P2* < 0.01 compared with the ischemia and hypoxia group

### TUNEL staining in the hippocampal CAl region

The number of apoptotic neurons and the AI were determined and calculated using TUNEL staining. As for the ischemia and hypoxia group, a lot of cells in hippocampal CAl tested positive to TUNEL staining, while cells in the rectal TH cooling group showed fewer positive neurons after TUNEL staining (Table 3).

**Table 3:** TUNEL positive cells and apoptosis index in hippocampus CA1 (mean ± SD, %)

Group	Number	Positive cell count (cells / × 400 field)	AI (%)
Healthy control	5	0	0
Ischemia and hypoxia group	10	23.2±7.7	26.6±12.1
Rectal TH cooling group	18	3.9±1.8	4.4±1.5

*P1* < 0.05 compared with the healthy control; *P2* < 0.05 compared with the ischemia and hypoxia group

## Discussion

It has been reported that the incidence of neonatal hypoxic-ischemic encephalopathy has reached 1.5/1000 [17, 18]. Several multi-center randomized controlled trials were carried out to evaluate the effects of TH induction (33.5–34.5 °C) in neonates (born at > 36 weeks’ gestational age) with moderate or severe hypoxic-ischemic encephalopathy, the results showed that TH treatment significantly decreased the mortality of newborns. Also, these studies found a lower incidence of neurodevelopmental disability in the TH-treated group; therefore, researchers have proposed treatment of hypoxic-ischemic encephalopathy with TH induction [6, 7, 18–28].

Though many cooling methods have been developed, their disadvantages still exist. The ice blanket is slow in decreasing the core body temperature, so its cooling efficiency cannot meet the requirement of diseases that need rapid TH induction. Besides, the ice blanket machine is too expensive for leading hospitals to afford, and the low mobility limits its application in pre-hospital emergency situations. Another cooling method, intravenous infusion of a low-temperature liquid, has shown high cooling efficiency when applied in clinical practice. It seems as an attractive strategy to achieve early cooling because of its portability, ease in administration, and potential widespread availability in the prehospital setting [12]. However, the indications, infusion liquid temperature, and the influence on vital signs, immune systems, hemodynamics and coagulation functions require verification; Though intravascular catheter cooling, extracorporeal blood cooling, and blood filtration can all achieve rapid TH induction, the requirement for vascular access placement may delay the treatment time and thereby affect the outcome of TH therapy [29–31]. What’s more, no studies have yet been published in which these devices have been used for longer-term (>24 h) cooling. Moreover, the above cooling methods have a great impact on the blood system, and, as a result, should be closely monitored by professional clinicians; which also hinders the promotion and application of TH therapy. The selective cerebral hypothermia method exerts a relatively small influence on systemic functions; consequently, it is more commonly used in cerebral resuscitation and brain protection for patients with emergent or severe neurosurgery injury [32]. However, this cooling strategy also requires further investigation regarding the direct monitoring of brain temperature, the selection of infusion vessels, the in vitro cooling circuit as well as the choice of infusion liquids.

The theoretical basis of this study is that ice-cold isotonic saline enema is a safe cooling method in fever patients, based on this, we proposed an optimal design for a disposable rectal TH cooling device. Two flow tubes are inserted into a closed storage bladder (elongated balloon) to realize a continuous cooling with isotonic ice-cold saline and animal’s core temperature is controlled by adjusting the rate of saline flow. This design achieved a satisfactory cooling efficiency; there was no direct contact between the saline and rectal mucosa, therefore, adverse reactions such as water intoxication could be well prevented.

Animal experiments showed that our rectal cooling device has achieved a higher efficiency of TH induction (37.3 ± 14.7 min) compared to the variable temperature blanket (75.6 ± 27.2 min). We also found that the temperature rise in the ice-blanket group at 330 mins. It may associated with shivering and heat generation. Furthermore, it also showed a favorable effect on SD rat protection and did not cause damage to the rectum of rats. Based on the Program of Hypothermia Treatment of Neonatal Hypoxic-Ischemic Encephalopathy (2001) [33], this study established a specific protocol for TH treatment using rectal cooling. This method could act as the basis for clinical application and promote the wide use of TH treatment.

There are some potential limitations of the current trial. First, the effect of brain protection in patients undergoing therapeutic hypothermia (TH) using ice-water circulating blanket has beeen confirmed by researchers, so we have no comparison between rectal TH and blanket TH rats using HE,NISSL and TUNEL staining.

Second, due to limited experimental conditions, we did not evaluate the vital signs of rats nor the long-term influence of TH treatment on rats’ neural system. Thus, the influence of this TH cooling device on rats’ vital signs and long-term recovery of neural functions are unknown. These potential limitations should be considered in the context of the trial’s strengths.

## Conclusion

1. Both the variable temperature blanket and our device achieved TH induction in rats with hypoxic-ischemic brain damage, but our device showed a higher cooling efficiency compared to the blanket.
2. Our rectal TH cooling device did not cause damage to rats’ rectal tissues.
3. TH treatment with our new device significantly decreased cell apoptosis in rats’ hippocampal CAl.
4. Our new cooling device is easy to carry and so might be applied in pre-hospital treatment and improve patients’ prognosis.

## Additional file

Additional file 1: Detail about rectal temperature in Rectal and Blanket cooling group. (XLS 28 kb)

## Abbreviations

TH: Theraputic hypothermia
